# Opportunistic Weather Sensing by Smart City Wireless Communication Networks

**DOI:** 10.3390/s24247901

**Published:** 2024-12-11

**Authors:** Jonatan Ostrometzky, Hagit Messer

**Affiliations:** School of Electrical Engineering, Tel Aviv University, P.O. Box 39040, Tel Aviv 6997801, Israel; messer@tauex.tau.ac.il

**Keywords:** smart city, ISAC, opportunistic ISAC, weather monitoring

## Abstract

This paper presents how the concept of opportunistic integrated sensing and communication (ISAC), focusing on weather sensing, is incorporated into wireless smart cities’ networks. The concept, first introduced in 2006, utilized standard signal level measurements from wireless backhaul cellular networks for rain monitoring. Since then, it has expanded to include technologies like satellite communication and smart cities’ networks. Opportunistic ISAC (OISAC) for weather involves transforming communication networks into virtual sensors by interpreting the signal attenuation caused by environmental factors, such as rain. These virtual sensors form the sensing layer of an IoT system, with built-in connectivity. In this paper, we present the recent advancements in the field, emphasizing the potential of current and future smart cities’ wireless networks for accurate rainfall monitoring. We also demonstrate a test case in the city of Rehovot in Israel, where high spatiotemporal resolution rain maps produced via the OISAC paradigm significantly outperform the spatial resolution achieved by modern weather radars. We also discuss the challenges and opportunities in applying this concept.

## 1. Introduction—Opportunistic Sensing of Weather

Weather monitoring, especially for precipitation, is crucial for modern applications like forecasting, water management, and smart city development. It also plays a role in understanding global phenomena like climate change and renewable energy potential. Within an urban environment, accurate rainfall monitoring is especially important for water management such as flash in-city flood prevention, and to help urban planners and engineers use rainfall information both to design efficient city infrastructure and better manage and control the city during extreme weather events scenarios. Traditional weather monitoring tools, such as rain gauges and weather stations, are precise but offer limited spatial coverage, while remote sensing technologies like satellites and radars provide wider coverage but are less accurate near the ground [1,2]. Moreover, the temporal resolution of remote sensing measurements is limited. Furthermore, installing and maintaining these systems is expensive and typically limited to developed countries, leaving regions such as Africa, Central Asia, and South America with poor or non-existing coverage. On the other hand, technological advancements use the atmosphere, and, in particular, the near-the-surface atmosphere, as a home for human-made electromagnetic radiation, for military and civilian uses. A few examples are radio transmission, cellular communication, satellite TV, and navigation systems. Nowadays, the electromagnetic spectrum is so crowded that in any given time and almost all around the globe, technology induced electromagnetic waves are present [3]. Naturally, the electromagnetic signals, traveling in the atmosphere in different frequencies, layers, and locations, carry information about the media they go through [4].

The idea of leveraging existing communication technologies for environmental monitoring, i.e., in an opportunistic use, is a relatively new and viable solution to improve environmental monitoring capabilities, as dedicated meteorological sensor network deployment involves high costs and expensive maintenance [5]. In [6], the Global Positioning System (GPS) was first used for water vapor sensing. Later, in 2006, it was first shown how commercial cellular backhaul networks can be used for rain monitoring [7,8,9]. This idea is based on utilizing the already deployed existing commercial wireless communication networks, and, in particular, commercial microwave and mmWave links (CMLs)—usually operating at frequencies above 6 GHz—for precipitation monitoring. The number of CMLs worldwide is immense (in order of millions of CMLs), and thus, adding this approach to the arsenal of weather monitoring can vastly help in reducing the need to deploy and maintain dedicated meteorological sensors. Specifically, in locations where dedicated meteorological equipment is lacking (e.g., Africa), cellular coverage indeed exists [10,11,12], further helping close the gap in the world-wide coverage of weather sensing.

In recent years, the concept of integrated sensing and communication (ISAC) started to gain interest as the next generation leading technology [13]. It was recently presented how high-frequency E-band-based CMLs can be used to monitor rain in urban environments [14,15] where traditional weather monitoring techniques often fail. The potential for weather opportunistic monitoring is further enhanced as future networks—and especially in urban environments—will become more diverse and include many line-of-sight microwave and mmWave links, all part of an xhaul mesh (combining the “classic” backhaul and fronthaul of wireless communication networks) that becomes a necessity for the modern city [16].

The ISAC (also known as joint communication and sensing—JCAS) paradigm aims at future technologies designed to optimally use the spectrum for both communication and sensing applications. It states that sensing capabilities (such as dedicated sensors) can be implemented as part of wireless communication nodes, and thus, combined equipment capable of both communication and sensing can be developed and deployed (e.g., wireless communication terminals with on-board temperature sensors) [17,18]. OISAC, on the other hand, is based on existing technologies, where the sensing function is added to communication (or vice versa). In our case, we utilize the wireless communication channel itself to sense the environment—especially the weather—opportunistically.

As stated, tens of research groups around the world have reported that CMLs as well as satellite microwave links (SMLs) are capable of accurately monitoring rain and other weather phenomena such as fog, humidity, and dew in many parts of the world (see [7,8,9,10,12,15,19,20,21,22,23] among many others), all falling into the definition of OISAC since it uses existing communication for sensing. In the following sections, we begin by reviewing the current state of OISAC for weather, especially detailing the use of CMLs, and the CMLs deployed in a dedicated installment for smart cities’ wireless networks (SCWNs). We then discuss and demonstrate the feasibility of using actual measurements for the potential of OISAC in smart city environments where SCWNs are integrated parts of every modern smart-city infrastructure. Lastly, we conclude this paper and discuss future directions and possibilities.

## 2. OISAC of Weather—Current Situation

**Figure 1:**
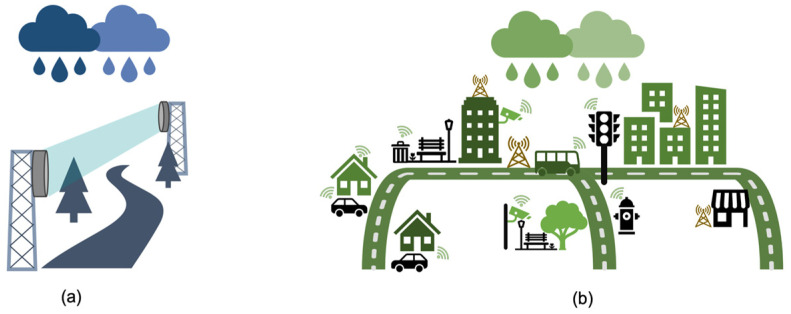
Opportunistic ISAC for weather using terrestrial wireless communication links, divided into two groups: (**a**) CMLs—point-to-point terrestrial microwave and mmWave channels (e.g., in cellular backhaul networks); (**b**) SCWNs—smart city wireless networks at mmWave frequencies (e.g., wireless communication mesh of smart IoT sensors at street level such as security cameras, traffic lights, bus stations, vehicles, and public facilities such as street trash cans and fire poles).

In recent years, many researchers in universities, in state-wise meteorological services, and in the private sector, dealt with different aspects of OISAC for weather; see, for example, EU COST Action OpenSense (https://opensenseaction.eu/, accessed on 25 September 2024), connecting more than 150 members from over 30 countries. When dealing with terrestrial communications, one can divide the communication into two groups: terrestrial CMLs used mainly in backhaul cellular networks, and SCWNs—for dense smart city connectivity. It is worth noting here that satellite communication usage for OISAC is also an evolving field [19,24,25]; however, in this paper we focus on near-ground weather monitoring. In Figure 1, we present an illustration of these two main groups, which emphasize their different deployment and application properties. Note that the division of these groups can be somewhat mixed, as in recent applications, especially under the IoT framework (and IoT in smart cities specifically), different technologies work in harmony as additional parts of a full SCWN, adding a diversity of opportunistic sensor-types, but on the other hand, introducing the challenges of working with different data-sources and devices, each with its own characteristics.

In general, an IoT framework consists of three layers [26,27,28]: The perception (or sensing) layer, the communication (or the connectivity) layer, and the processing (or the data) layer. In the ISAC paradigm for IoT, the sensing capabilities (which corresponds to the end-user hardware itself, such as the transmitted/receiver modules, e.g., with additional hardware aimed for sensing phenomena of interest) are integrated with the communication layer (for further background details regarding OISAC and IoT, the reader is referred to Supplementary Materials).

The OISAC approach is used to add the sensing of the weather to the existing communication layer. In smart cities, where a vast number of connected devices are available, the number of channels is high, which raises the potential for high spatiotemporal weather products (such as rain-field maps [14,29]).

**Figure 2:**
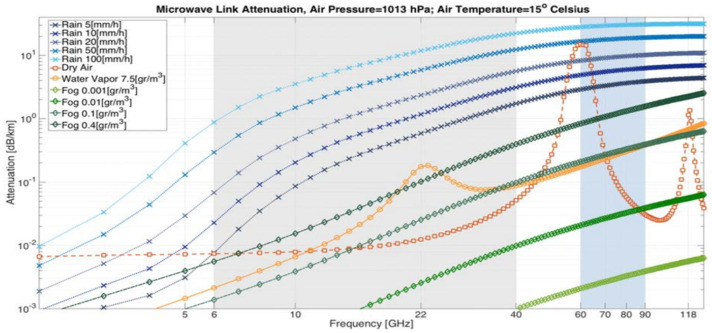
The expected signal attenuation (in dB/km) as a function of the channels frequency due to rain, water vapor, and fog. This image is taken from [30] and is based on the ITU recommendation P.838-3 [31].

We focus on wireless communication in the microwave and mmWave frequency bands (i.e., 5 GHz to 100 GHz), as the signal level in these frequencies is more affected by weather, as shown in Figure 2. Luckily, this frequency range is the one used mainly in the backhaul CMLs and also in modern fronthaul and xhaul CMLs and SCWNs.

### Commercial Microwave and mmWave Links

The OISAC of the weather using wireless links as sensors is based on the fact that the attenuation levels of each of the links are being logged (usually automatically by the network management systems, for network management purposes), and can be converted into a time series of, e.g., rain-intensity values, as if it was reported via virtual rain gauges, based on the relationship between the rain-induced attenuation and the actual rainfall. In general, the relationship between the channel rain-induced attenuation and the rain intensity is usually modeled by an empirical power law. It is an approximation that gives a reasonably accurate relationship for links of the length of hundreds of meters and above (up to few kilometers) in micro and mmWave frequencies. This power-law is given by [32]

(1) $$A=a{\underline{R}}^{b}$$

where A is the rain-induced attenuation per kilometer (in dB/km), $\underline{R}$ (in mm/h) is the path-averaged instantaneous rain rate along the link, and {a,b} are coefficients whose values depend on the link’s frequency, polarization, and on the local drop size distribution (i.e., the local climatological conditions) of the rain [31]. That is, given the measured attenuation in a CML and the coefficients {a,b} that were derived for the various areas, $\underline{R}$ can be directly extracted and each CML can be transformed into a virtual rain gauge. Moreover, in [33] it was proposed that, given several CMLs in an area, each CML can be transformed into several virtual rain gauges.

It is worth noting that the power law of Equation (1) approximates the full physical description of the interaction between water droplets and the signal, which is based mainly on Mie scattering (see [34] for the full development of the formula). The coefficients {a,b}, although set empirically [31], are known to produce accurate CML-based rain estimates in many parts of the world, including Africa [12], Europe (Germany [21,23], Sweden [35], the Netherlands [22], the Czech Republic [20]), and the Middle East (Israel [30,33]) among others.

In practical use, as several factors can cause attenuation to wireless microwave signals, as illustrated in Figure 2, the first step in this model-driven approach is to isolate the rain-induced attenuation. The total attenuation of the wireless signal at a line-of-sight link of length L (in km) at time t, $A_{T}\left(t,L\right)\;\left(in\;dB\right)$ can be expressed as follows [36]:

(2) $$A_{T}\left(t,L\right)=A_{0}\left(L\right)+A_{m}\left(t,L\right)+A_{p}\left(t,L\right)+A_{w}\left(t\right)+A_{n}\left(t\right)$$

where $A_{0}\left(L\right)$ is the link path loss, $A_{m}\left(t,L\right)$ is the contribution of moisture (water vapor), $A_{p}\left(t,L\right)$ is a component which represents the contribution of precipitation (if exists), $A_{w}\left(t\right)$ is the contribution of the wet antenna effect, and $A_{n}\left(t\right)$ represents all the other sources, defined as “noise”, such as the obstacles that may be present at the propagation channel (e.g., birds [37]), and the measurement noise. For simplicity, in the sequel, we assume that precipitation is pure rain, so the term $A_{p}\left(t,L\right)\equiv A_{L}\left(t\right)$ represents the rain induced attenuation.

Extracting the rain-induced attenuation from the signal level measurements provided by the cellular operators is far from being trivial. First, $A_{T}\left(t,L\right)$ in (2) is not directly measured and is to be extracted from the available measurements of the transmitted (Tx) and the received (Rx) signals levels, which are usually quantized and are not synchronized. Then, the rain-induced component is to be extracted from $A_{T}\left(t,L\right)$ by filtering other-than-rain components from the overall attenuation (many methods for performing it have been proposed, e.g., [20,30,38,39] among many others).

An alterative approach for transforming signal level measurements in CMLs to rain measurements is a data-driven one. As the amount of data collected by CMLs has become sufficient (in the sense of sufficient historic data and climatological diversity), data-driven approaches have become possible, and several algorithms to do so have been presented (e.g., [21,40,41,42], to name a few). Moreover, some data sets are even freely available online [35,43,44], which encourages developing more data-driven algorithms for the processing layer in the CML-based IoT for weather monitoring. Given the wide spread of CMLs, once each is converted to at least a single virtual rain gauge, it is possible to produce a number of rain products, mainly wide range rain maps at a resolution of at least the standard weather radars, and often better [22,23].

Lastly, it is worth mentioning that although rain is the major factor that causes attenuation in wireless signals at the K-band and the E-band frequency ranges, other-than-rain water-based phenomena can also be monitored via the proposed OISAC approach. A few examples are as follows: humidity [45], dew [36], fog [46] retrieval and detection [47], and the estimation of accumulated mixed precipitation (e.g., snow, rain, and sleet) [48].

## 3. OISAC in Smart Cities

Smart cities have come a long way in terms of infrastructure in recent years. Current and future smart cities rely heavily on wireless communication channels operating at mmWave frequencies and, as such, can be utilized as opportunistic weather sensors. Anything from low-power communication devices (such as sensor reporting and utilizing services for citizens and the management of the city, e.g., full trash cans, bus arrivals, traffic light statuses, etc.), to high-bandwidth communication links for autonomous vehicles and street cameras are all interconnected in a vast xhaul line-of-sight grid. Future mobile devices (5G and above) will also implement mmWave modems, giving even more diversity of channels in dense connected urban environments.

Until a few years ago, CML networks were mainly used for the purpose of establishing a stable and static backhaul of cellular networks and indeed, 3G and 4G cellular networks rely heavily on such CMLs at frequencies in the K-band (6–40 GHz). In recent years, as the need for a larger bandwidth arose, higher frequencies in the E-band wireless links started to be deployed. Moreover, such E-band CMLs offer a higher throughput, and thus, are a good solution for the city-wide networks used for designated smart-city applications such as street camera feeds, public transportation real-time control, Wi-Fi services to the residents, etc. As the carrier frequency increases, it is obvious that E-band links’ capacity is higher than K-band links, but at the cost of higher propagation losses of the signal. Therefore, E-band CMLs are generally shorter—a fact that by itself introduces additional challenges for opportunistic rain monitoring—from additional non-negligible other-than-rain attenuation phenomena such as wet-antenna effects [49,50] through the general phenomena of overestimation of the rain intensity when using the standard power law (of Equation (1)) [29]. On the other hand, these frequencies are more sensitive to precipitation (See Figure 2). Recent studies showed, however, that SCWN can be exploited for opportunistically generating rain measurements that, in turn, lead to accurate near ground spatiotemporal rain maps [51]. It can also be used for the near-real time estimation of storm front movement [52], a capability that is of the utmost important for real-time city management and security service responses, especially during heavy and extreme weather events in cities.

**Figure 3:**
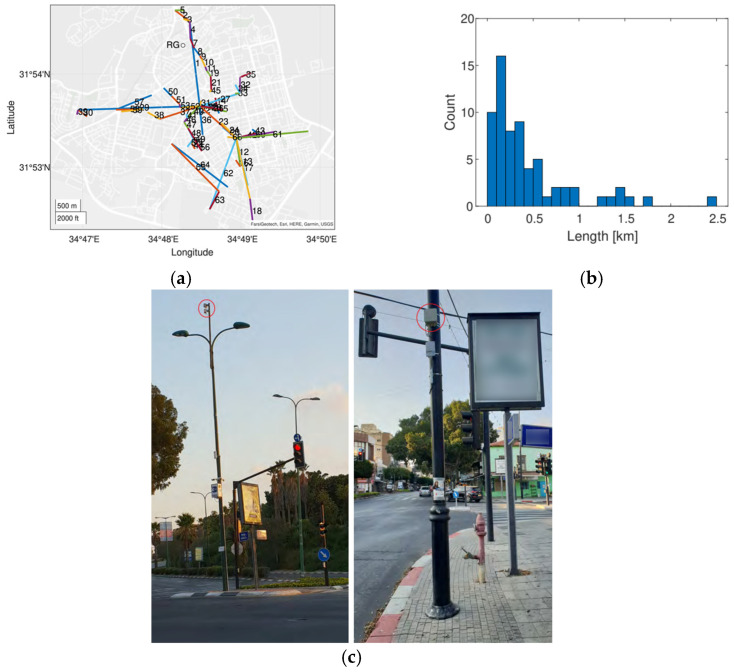
(**a**) A map of the city of Rehovot, Central District, Israel, with 66 SCWN’s links (marked by colored lines and numbering 1 through 66) operating at a frequency range of 70–80 GHz. The shortest link is 53 m, and the longest is 2.42 km [53]. (**b**) The 66 SCWNs’ path length histograms [53]. (**c**) An image of an installation of two Tx/Rx terminals which form the nodes of the SCWNs’ networks. Each of the terminals is marked via a red circle [54].

**Figure 4:**
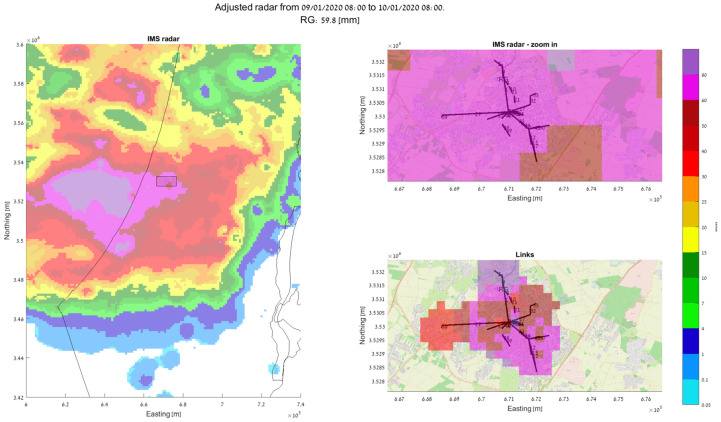
Accumulated rain during a 24 h period of heavy rain (total rainfall: 59.8 mm). **Left**: IMS weather radar rain map. **Top right**: zoomed-in radar map, covering the city of Rehovot (of Figure 3). **Bottom right**: a map generated by the available SCWN’s links [53].

**Figure 5:**
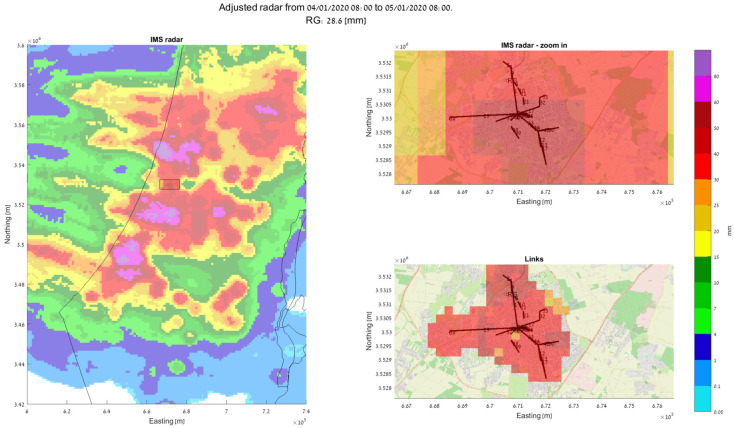
Accumulated rain during 24 h period of moderate rain (total rainfall: 28.6 mm). **Left**: IMS weather radar rain map. **Top right**: zoomed-in radar map, covering the city of Rehovot (of Figure 3). **Bottom right**: a map generated by the available SCWN’s links [53].

**Figure 6:**
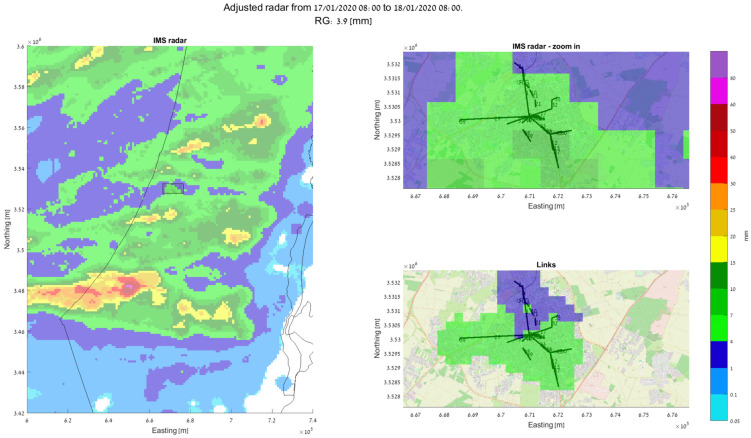
Accumulated rain during 24 h period of light rain (total rainfall: 3.9 mm). **Left**: IMS weather radar rain map. **Top right**: zoomed-in radar map, covering the city of Rehovot (of Figure 3). **Bottom right**: a map generated by the available SCWN links [53].

Based on our recent works [14,53], we present a demonstration of how using signal-level measurements from an actual SCWN improves the spatiotemporal resolution of rain monitoring in urban environment significantly. In Figure 3a, a setup of an actual SCWN in the city of Rehovot in the central district of Israel is depicted. There, a total of 66 links at frequencies of 70 GHz to 80 GHz are deployed at an area of about 10 km × 10 km. The shortest link in this network has a length of only 53 m, and the longest one spans over 2.4 km [53]. A histogram of all the SCWNs’ path lengths is depicted in Figure 3b [53], and an image of the actual Tx/Rx terminals is presented in Figure 3c (taken from [54]). The full hardware description of the terminals is available in [54]. Overall, most of the links are shorter than 1 km. As the power law (of Equation (1)) was empirically proven to be problematic in terms of its accurate rain intensity estimation when dealing with short links [55], we performed a calibration of the power law, by using the longest available link as a calibration reference [29] (as longer links are not affected by the short-link inaccuracy of the power law). In Figure 4, Figure 5 and Figure 6, the full estimation workflow’s results are presented, during three days in which rain occurred—a heavy rain event (59.8 mm within 24 h), a moderate rain event (28.6 mm within 24 h), and a light rain event (3.9 mm within 24 h). In each of these figures, on the left, one can see the 24 h accumulated rainfall (in mm) as was reported by the Israeli Meteorological Services (IMS) radar, with a zoomed-in section of the map—of the city of Rehovot—depicted on the top right of the figure. At the bottom right corner of the figure, one can see the same map of accumulated rain—as was estimated by the proposed approach from signal level measurements in the SCWN. As one can see, the spatial resolution reported from the SCWN was higher than the resolution achieved by the radar by an order of magnitude: the radar spatial resolution in this scenario was 1 km by 1 km (based on the IMS radar data), whereas the SCWN produced a snapshot with a spatial resolution of roughly 300 m by 300 m. This high resolution will help smart cities’ management systems in the future to better operate their cities—including water management, risk assessment, public and private transportation needs during and after weather events, electricity distribution in a smart-grid, etc. It is also worth mentioning that whereas the radar produces a single map once every 5 to 15 min (a standard value range for weather radars world-wide), the links produce a channel attenuation value every 30 s. That is, the temporal resolution of the SCWN-based rain products are also higher by an order of magnitude when compared to the standard weather radar, opening the options for actual real-time weather-influenced city management.

## 4. Open Issues in Opportunistic ISAC and IoT

### 4.1. Non-Attenuation Measurements

Currently, the overwhelming majority of OISAC for weather is based on the measured signal level in wireless microwave and mmWave channels. Different from the traditional IoT concept, in which designated sensors are connected by wireless systems in order to allow for weather monitoring [56,57,58], the concept of OISAC is to use the already available measurements that are being collected for communication purposes, without the burden of requiring any additional hardware. As long as the signal’s Tx and Rx (or Es/No when dealing with satellite terminals) values were the main standard measured values (either instantaneous, average, or minimum and maximum values per specific time intervals), extracting weather information from such measurements was the main challenge.

Emerging wireless technologies of both CMLs and SCWNs will divert from the classic “division” of backhaul and fronthaul. For example, integrated access and backhaul [59] allows the use of phased array antenna modules to either communicate with mobile devices, or to create, in real-time, an ad hoc backhaul channel between two (or more) modules, in order to enhance the wireless network where needed (e.g., during sport events where a specific location requires additional bandwidth capabilities, or during a malfunction or unavailability of a network node that creates the need for diversion of the data around it). Such antenna arrays, as well as other capacity enhancement technologies such as MIMO cannot be controlled and managed via the attenuation values alone. Thus, future devices that build such xhaul wireless networks will report additional information such as the full channel modulation phase between different antennas in the array regularly, and the latency between the transmitted signal and the received one, which hold additional information that can be exploited for opportunistic weather monitoring purposes (e.g., [60,61]). Furthermore, to enhance the network redundancy, some hardware manufacturers propose a multi-band [62] approach, in which a number of CMLs share the same path using different frequencies, increasing the amount of the available data and its diversity. The use of additional information can be explored and exploited, as such data have already been shown to include additional information about the weather. In a way, it can be viewed as analogous to the optical readings made by a disdrometer, capable of reporting the type of fallen precipitation and the drop size distribution with high accuracy using a set of light emitting diodes or lasers diodes to create an optical plate passing through the air and used to detect precipitation particles [63]. In addition, the number of available transmitters and receivers is expected to increase exponentially, as 5G and xhaul SCWNs will penetrate our daily lives [64,65].

Looking back at Figure 1b, an illustration of future SCWNs can be seen. This features a futuristic IoT network including many devices, all connected by mmWave signals: bus stops, trash cans, electric vehicles charging stations, etc. As future smart cities will rely on heavy IoT connected devices [65,66], the number of available OISAC data sources is almost limitless. All the data about the signal quality of such devices (or even more primitively, the connection status of such devices, which can inform us about “good/bad channel properties”) can be processed either at the edge, or in a centralized location. Furthermore, the all-time connectivity of the devices in the new smart city scape could be implemented further, as recently shown in [67], where air temperature trends were extracted from smartphones built in sensors data.

### 4.2. Wireless Networks Quality of Service Optimization

Up to this point, we presented how OISAC can be used for weather monitoring, specifically, in the smart-city scene. However, one can think in the opposite direction, using opportunistic sensitivity to improve communication. As weather phenomena influence the performance of communication networks, and since CMLs and SCWNs report attenuation (and other signal-based data) back for network management purposes, it is possible to add an additional layer of processing, which could detect disturbances in the network throughput in real-time or near real-time, and use these disturbances to predict future disturbances. In a recent work [68], we presented how one can detect such disturbances (caused by a storm) and predict these disturbances due to the storm movement up to a few minutes ahead of time. Such predictions can make the network management proactive (e.g., by changing the network switching and rerouting profiles ahead of time), and thus, improve the quality of service (QoS) of the network dramatically.

## 5. Summary and Conclusions

High temporal–spatial resolution weather monitoring on the ground level is essential for many human applications. Within an urban environment, accurate rainfall monitoring is especially important both for smart city planning and for its operation.

Such high-resolution monitoring can be achieved by a dense network of designated sensors. Such a solution, however, is unrealistic because of practical deployment and maintenance challenges. Moreover, because of the local climate created in cities, remote sensing techniques for weather monitoring are less effective in urban areas [69]. In this paper, we presented the paradigm of using opportunistic sensing of the weather using the wireless communication infrastructure that is present in modern smart cities’ infrastructure. The idea is to use the already available hardware that produces standard attenuation measurements in SCWN as virtual weather monitoring devices. As such, they can be viewed as OISAC devices with no (or negligible) additional cost. The performance of the weather products depends on the availability of sensors and their spread. Naturally, the density of the virtual sensors is proportional to the density of the population in a given area. Here, smart cities provide a win–win situation, as the denser the population is, the higher the number of microwave and mmWave-connected devices are available. As a demonstration in the city of Rehovot in Israel, a rainfall map with a spatial resolution of an order of magnitude higher than the standard weather radar has been achieved.

Lastly, we emphasize that OISAC is not only confined to rain monitoring. Indeed, monitoring different weather phenomena poses additional challenges, but past studies established the potential of using CMLs for not only near-ground rain mapping (e.g., [22,70]), but also for many other weather phenomena [71]. Thus, we conclude with emphasizing that the concept of OISAC should be integrated into the design and management phases of modern smart cities, as it has the potential to benefit them greatly.

## Data Availability

Data are contained within the article and supplementary materials.

## Supplementary Materials

Part of the ideas presented in this paper are described in the IEEE ISAC FOCUS newsletter (Issue 6, 2023) online at https://isac.committees.comsoc.org/wp-content/uploads/sites/147/2023/05/ISAC-Focus_Issue6_0520.pdf (accessed on 25 September 2024).
